# Structural, Optical, and Electrical Properties of Hafnium–Aluminum–Zinc-Oxide Films Grown by Atomic Layer Deposition for TCO Applications

**DOI:** 10.1021/acsomega.3c04256

**Published:** 2023-08-09

**Authors:** Maciej Krajewski, Mateusz Tokarczyk, Piotr Świętochowski, Piotr Wróbel, Maria Kamińska, Aneta Drabińska

**Affiliations:** Faculty of Physics, University of Warsaw, 02-093 Warsaw, Poland

## Abstract

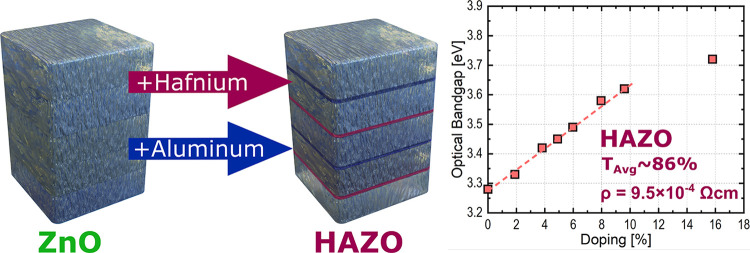

ZnO is a widely studied material that exhibits versatile doping possibilities. Most research presents singly doped ZnO, leaving the potential of codoping unexplored. Within this study, hafnium–aluminum codoped zinc oxide (HAZO) thin films were grown on a glass substrate using the atomic layer deposition technique at 200 °C. A comprehensive analysis of the surface morphology and electrical and optical properties of the samples was conducted for varying the Al/Hf doping ratio. X-ray diffraction studies showed that the obtained films are polycrystalline, exhibiting a preferential growth direction along the (1 0 0) plane without any detectable precipitates. Moreover, the electrical measurements of HAZO films revealed that they exhibit lower resistivity (∼9.5 × 10^–4^ Ωcm) than the commonly used aluminum zinc oxide films (AZO). This improvement can be primarily attributed to the promotion of the n-type carrier concentration to 4.45 × 10^20^ cm^–3^ while maintaining a mobility value equal to 14.7 cm^2^/Vs. The doping also influences the optical properties of the material by widening the band gap and changing the refractive index, as observed by spectroscopy and ellipsometry studies. These findings highlight the potential of proposed HAZO thin films for future applications in electronic devices utilizing transparent conducting oxides.

## 1 Introduction

In recent decades, there has been increasing interest in transparent conductive oxide (TCO) films such as In_2_O_3_, SnO_2_, ZnO, and their alloys variations, primarily due to their potential applications in electronic and optoelectronic devices including solar cells, LEDs, flat panel displays, thin film transistors, and variety of sensors.^1−8^ TCOs are characterized by having simultaneously exceptional electrical conductivity and high transmission in the visible range. These properties of the TCO films can be widely tuned by changing various factors such as their growth parameters, composition, doping amount, and post-deposition treatment.^9−15^ Among many TCO materials, doped ZnO is one of the foremost candidates for next-generation electronic devices, due to its high earth abundance, nontoxicity, biocompatibility, and affordability, contrary to current industry standard indium tin oxide (ITO).^16−18^

ZnO is an n-type intrinsic semiconductor with a direct and wide band gap (∼3.37 eV), high carrier concentration exceeding 10^19^ cm^–3^, and very low resistivity in the range of 10^–3^ Ωcm.^19^ The electrical properties of ZnO may be further enhanced and tuned by increasing the carrier concentration, widening the band gap, and lowering electron affinity.^20−24^ This is usually done by adding doping elements to create an oxide alloy. Many reports show successful doping in low concentration regimes (below 7%) of metals from the IIIA group, such as Al, Ga, and In,^25−30^ transition metals Ag, Cu, Mn^31−33^ and even rare-earth elements such as Ce and Er^34,35^ where the introduced metal substitutes the zinc atom in the structure, leading to an additional ionized electron in the conduction band. Moreover, some reports investigate doping ZnO with IVB elements such as Hf, Ti, and Zr,^36−42^ which enhances the material’s electrical stability, modifies the conduction band minimum, and fills the oxygen vacancies states. While the properties of singly doped ZnO have been extensively studied in numerous studies, there still remains undiscovered potential in codoping ZnO with two elements simultaneously. This approach can lead to the formation of quaternary material with novel optoelectronic properties, offering new pathways for exploration and applications.

For example, Kang et al.^43^ have shown that Al/F codoping passivates trapping defects (e.g., V_O_) and thus enhance the mobility of the sample obtaining remarkably low resistivity equal to 5 × 10^–4^ Ωcm. Also, pairs such as Al/Sn, Al/Ti, Al/Th, or Al/Mn were reported to exhibit exceptional optoelectrical parameters, offering one additional degree of freedom in material design. Research findings also indicate beneficial results of codoping using two elements from different groups, such as IIIA and IVB.^43−50^ Combining elements from those two groups may lead to enhanced electrical stability and further promote the mobility and concentration of the carriers in the material.

The above-mentioned quaternary materials can be grown by a vast variety of techniques, including sol–gel, CVD, sputtering, or atomic layer deposition (ALD). The samples with the lowest resistivities, reaching as low as ∼2 to 4 × 10^–4^ Ω cm, are usually obtained with RF sputtering and in a H_2_-rich atmosphere enhancing free carrier formation due to hydrogen-related defects.^51−53^ Although promising for enhancing electrical properties, H shallow donors are reported to be unstable and thus prone to diffusing out even in moderate temperatures, leading to long-term electrical instability of the material.^53−55^ Another well-adopted deposition technique for ZnO thin films is ALD. It offers precise control of the film thickness due to the self-limiting character of the reactions during every half-cycle. In particular, it allows precise control over the composition of deposited material due to the pulse and purge repetition cycle in which precursor vapors penetrate the sample. Therefore, this technique is used to uniformly coat sophistically engineered structures with a high-aspect ratio, which remains unreachable for sputtering techniques.

In this study, we fabricated codoped ZnO films by combining elements from IIIA and IVB groups, i.e., quaternary hafnium–aluminum zinc oxide films (HAZO), utilizing the ALD technique. While aluminum is already widely used in ZnO structures (AZO) as an electron dopant, hafnium is a novel element introduced to this material. Hafnium in the ZnO structure is therefore an oxygen vacancies suppressor, stabilizing the whole lattice and giving additional electrons to the conduction band, as well as helps to mitigate defects present on the grain boundaries. The filling of those defects within the oxide should therefore lead to a shift in the maximum achievable free carrier concentration and hinders mobility reduction. In this study, the surface morphology and optical and electrical properties were investigated over a wide range of dopant concentrations to optimize the structure. To the best of our knowledge, this is the first successful demonstration of mixing those two dopants within the ZnO lattice using the ALD technique, resulting in an exceptionally conductive and transparent material. The proposed codoped zinc oxide can find applications in future electronic devices utilizing TCO materials.

## 2 Results and Discussion

### 2.1 Morphology Characterization

**Figure 1:**
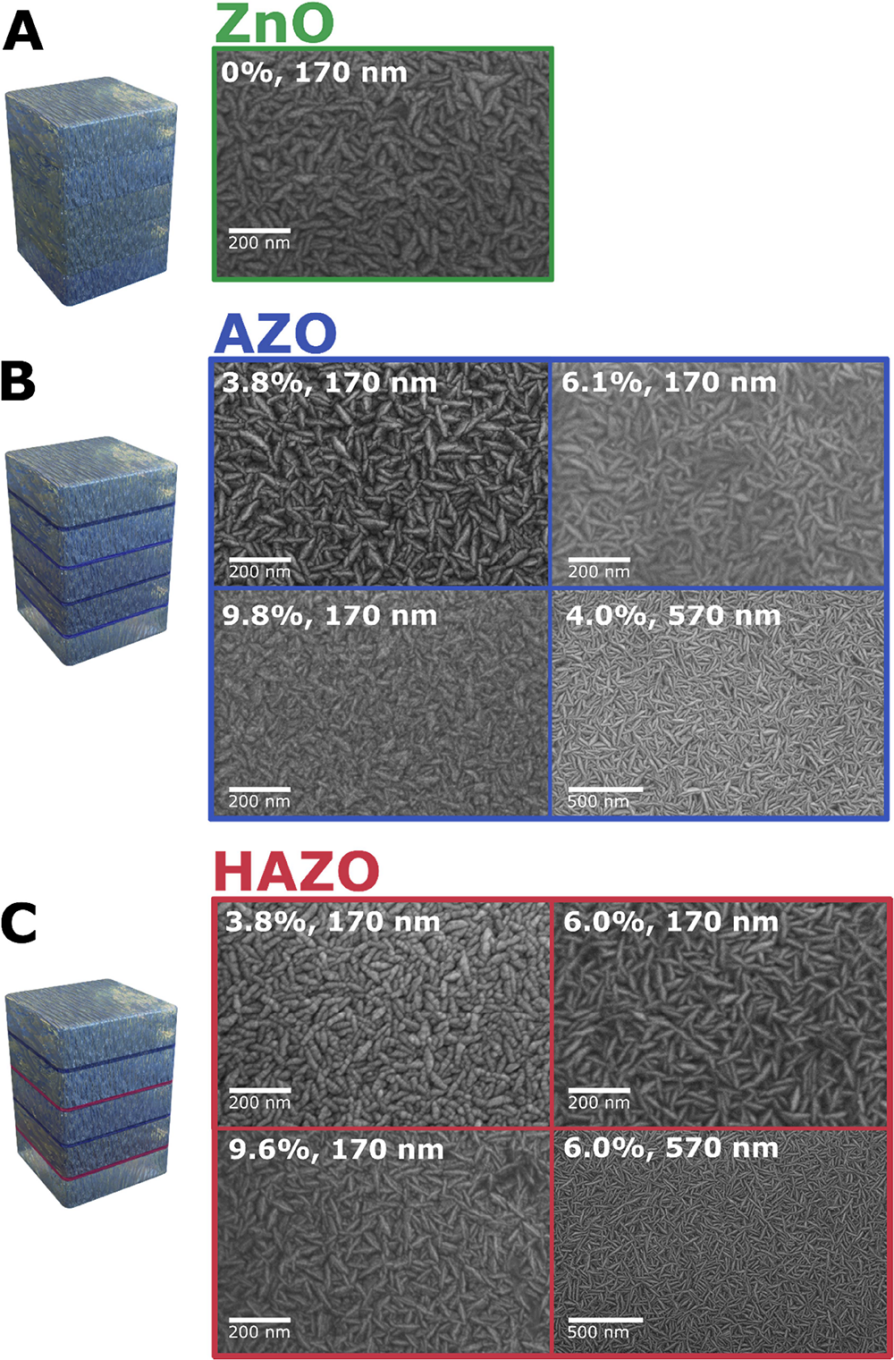
SEM images of (A–C) ZnO, AZO, and HAZO samples (170 and 570 nm thickness) with different dopant contents. On the left, the schematic drawings of the sample structure are shown, depicting the dopant relative positions during growth (aluminum as blue and hafnium as red stripes).

Thin film samples of both AZO and HAZO were grown with varied doping ratios as high as 10% of total precursor pulses number to compare their optoelectrical properties. In all cases, HAZO samples were doped with the same amount of each dopant, e.g., 6% HAZO means doping with 3% of aluminum and 3% of hafnium. SEM images of the 170 and 570 nm thick thin film samples’ surface for selected dopant concentrations are presented in Figure 1. Also, the schemes of the dopants present in samples are shown on the left of the SEM images. Note that the dopants, due to inter-diffusion, are uniformly or close to uniformly distributed in the structure and do not create planar layers; therefore, the drawings should be treated as a scheme of the pulses sequence during the growth. As can be seen, the 170 nm thick samples exhibit similar grain structure, resembling elongated double wedges with around ∼50 nm in length. As a result, a tight-packed landscape with disordered grains is formed.

**Figure 2:**
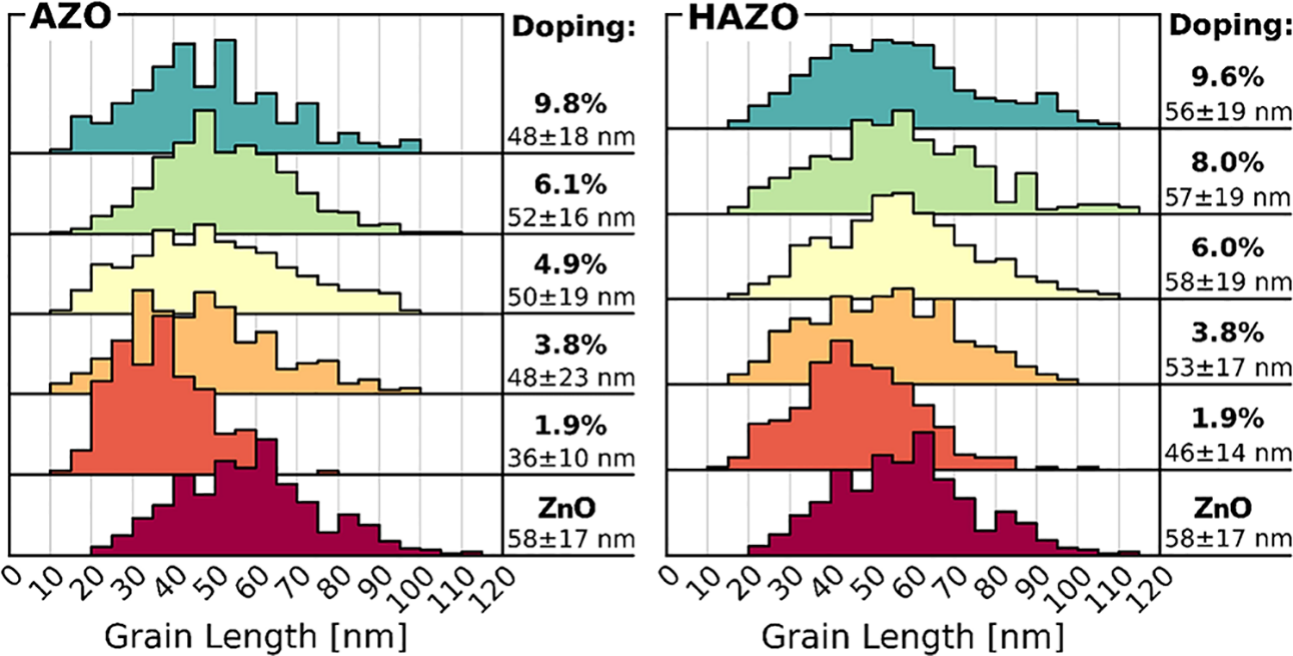
Granulometry results obtained from SEM images for both types of dopant samples with varying concentrations. On the right of each histogram, the average value of the grain length along with the standard deviation is presented.

The ZnO thin films commonly exhibit vertically preferred growth, which may result in differences between crystallite sizes in vertical and horizontal directions. Herein, the horizontal grain sizes visible in SEM images are investigated. The average grain size value distributions are depicted in Figure 2, with both materials demonstrating consistently stable grain length throughout the studied dopant concentration. The pure ZnO sample presents an average grain length of approximately 58 nm, slightly higher than the AZO and HAZO samples which exhibit an average of 48–52 and 55–57 nm, respectively. The size distribution of AZO grains is slightly more varied, showing a relative standard deviation ranging from 28% to even 48%, whereas the HAZO samples demonstrate lower variance in grain sizes, with a relative standard deviation in the 30–34% range. At higher dopant concentration (∼10%), this structure starts to be slightly more amorphic, in particular, in the case of AZO.

Those morphological changes visible in the strongly doped samples may be attributed to the critical stress reached in the wurtzite structure where no more dopants can be incorporated into the intrinsically available crystal sites. This results in the appearance of inclusions and a general deterioration of the crystalline quality leading to a decrease in Hall mobility. In the case of thicker samples (Figure 1D), the grains are notably longer, reaching around 100 nm.

**Figure 3:**
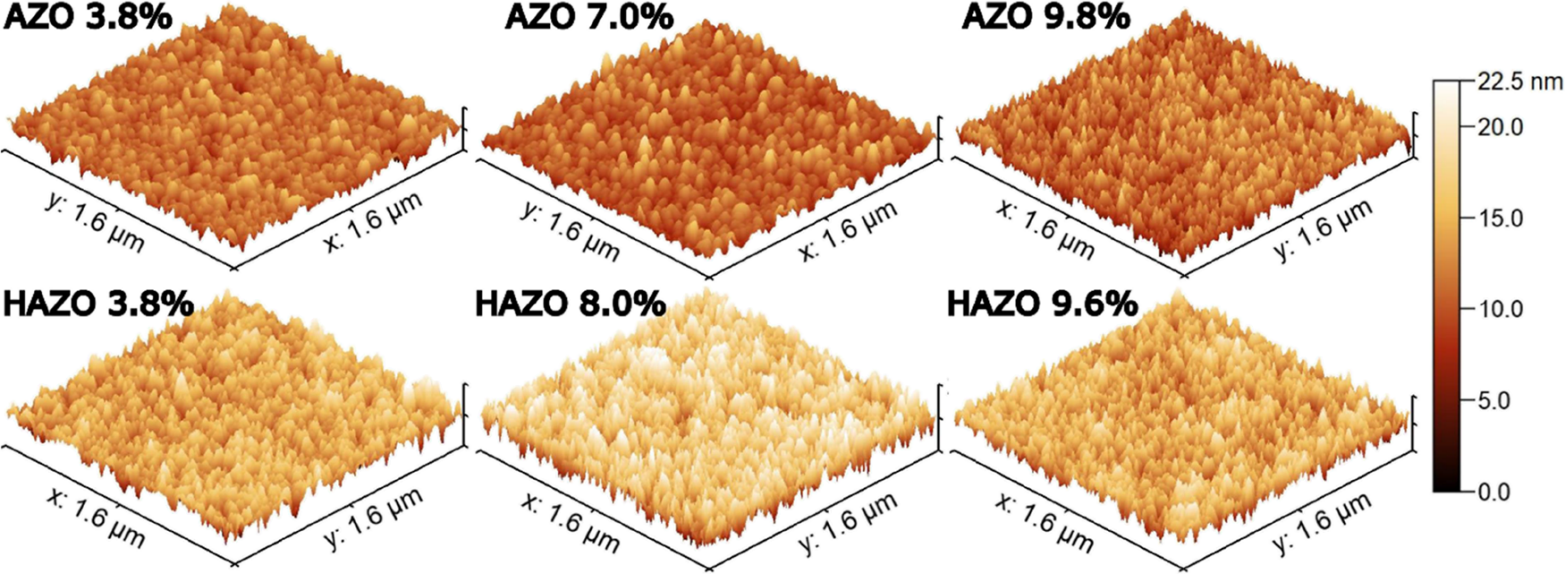
AFM 3D images showing the surface morphology of each sample with different dopant concentrations.

The samples’ morphology was further investigated by atomic force microscopy (Figure 3). The surface roughness exhibits only slight differences and does not follow a monotonic trend with respect to the dopant concentration. The grain sizes are correlated to the dopant percentage that suggests that the roughness may be also influenced by their organization patterns such as their steepness and directional alignment.

The root mean square (RMS) values of 2.0–2.3 and 2.7–3.4 nm were obtained for AZO and HAZO, respectively. These values are similar to those found in the literature.^56,57^ Seemingly, the hafnium addition affects slightly the surface roughness, making it more noticeable. Nevertheless, the AFM studies reveal that below 10% doping of hafnium, the surface is uniform and smooth. This indicates that the hafnium is incorporated similarly to aluminum, rather than forming nonstoichiometric aggregates and precipitates. Such formations may lead to crystal growth suppression and result in smaller grains with more pronounced boundary surfaces.

### 2.2 XRD Results

**Figure 4:**
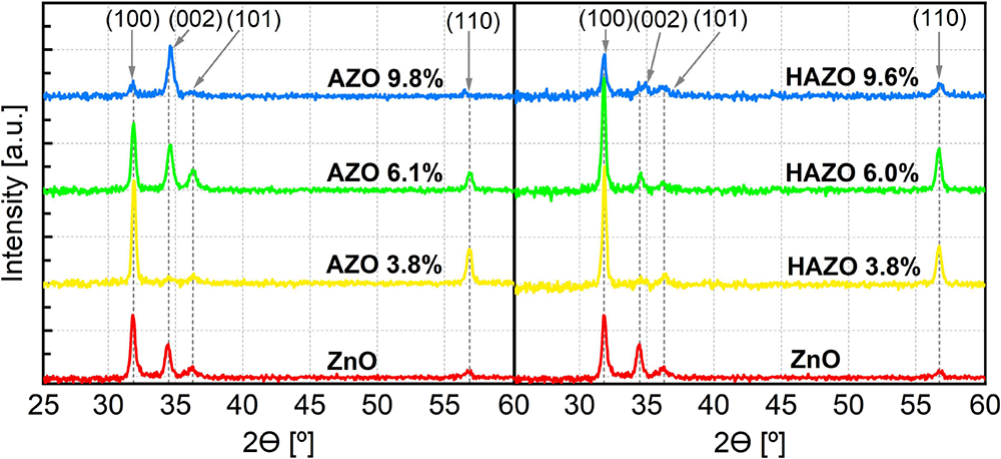
X-ray diffractograms of as deposited AZO (left) and HAZO (right) selected thin films on the glass substrates with a different dopant content. ZnO results are shown as the reference.

X-ray diffraction was utilized to investigate the crystalline structure of the materials. Figure 4 presents the diffractograms of the AZO- and HAZO-selected thin film samples with varying doping ratios. All films exhibit polycrystalline nature with a hexagonal wurtzite-type structure. Notably, no peaks associated with various phases such as Al_2_O_3_, HfO_2_, or others were detected, suggesting that codoping does not alter the ZnO wurtzite structure. The diffraction peak positions are in agreement with the reference pattern taken from JCP2.2CA 01-079-2205.

**Figure 5:**
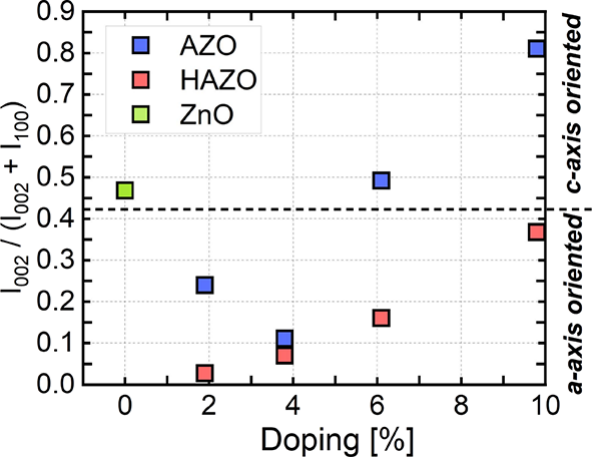
Intensity relationship between (002) and (100) peaks in AZO and HAZO samples showing the preferred orientation of the crystallites in the thin film. The dotted line represents the theoretical threshold for either *a*- or *c*-axis oriented sample. Pure ZnO is marked as a green square.

The presence and relative intensity of the (100) and (002) peaks are indicative of the preferential growth direction and gives information about crystallite shapes.^58,59^ The (100) peak is related to the *a*-axis preferred crystallites, whereas the (002) is related to *c*-axis-oriented growth (Figure 5). The introduction of either Al or Hf dopant does not cause a substantial shift in the peak positions, as they remain very close to their initial ZnO position even at high doping ratios. The small shifting visible in (002) and (110) peaks at 6.1 and 9.8% doping for AZO sample may be attributed to the very slight lattice changes. Usually, the incorporation of dopants smaller than host atoms, like Al to the ZnO structure (Zn^2+^ atomic radius is equal to 0.74 Å while Al^3+^ atomic radius is equal to 0.53 Å) leads to compressive stress and thus potential lattice constant changes. In the case of HAZO, except Al^3+^ ions, there are also Hf^4+^ ions present with an atomic radius very close to that of Zn^2+^ (Hf ^4+^ – 0.78 Å), which theoretically should not cause significant lattice alterations. However, as stated earlier, the XRD results did not reveal any substantial (more than 1% lattice constant compression) changes upon doping. These findings are consistent with those of other research studies on ALD-grown doped ZnO thin films, where notable alterations in lattice parameters were not observed.^44,57^ The variation in the intensity of these peaks leads to the dominance of different growth orientations depending on the dopant concentration and the material. For AZO, the (002) peak is dominating the whole spectrum above 6% of doping. On the other hand, the HAZO samples keep the *a*-axis preferential growth, which is visible by the prominent (100) peak in the spectrum.

This observation highlights the main distinction between the samples, suggesting that the Hf addition shifts the preferred growth orientation from the *c*-axis (as observed in pure ZnO) to the *a*-axis. A similar phenomenon was observed in ZnO–Ti,^60^ where Ti^4+^ ions may disturb the charge distribution of the (100) plane, in consequence lowering its surface energy and making it therefore the favored growth plane. Furthermore, the absolute intensity of the peaks is attenuated with the higher dopant addition, implying an ongoing amorphization process. This is consistent with the SEM images and electrical measurements, which also indicate the deterioration of the crystalline quality for high dopant concentrations.

### 2.3 Electrical Properties

**Figure 6:**
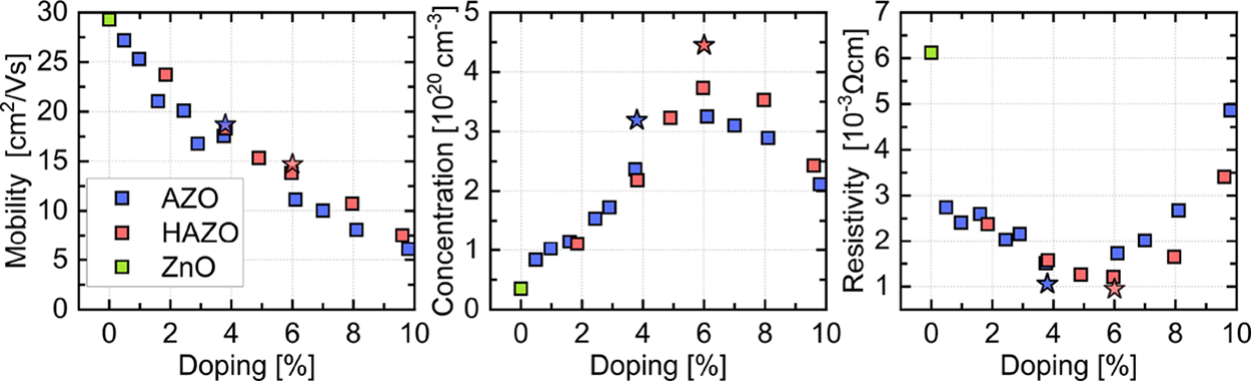
Electrical parameters of the studied materials for varying cation metal contents. Pure ZnO sample (i.e., with 0% doping) is indicated with a green square. The stars indicate the 570 nm AZO (blue) and HAZO (red) samples.

Figure 6 shows the electrical parameters (mobility, concentration, and resistivity) of the studied materials as a dopant amount function. Table 1 summarizes them in the selected samples with the same doping content as shown in the previous figures. As anticipated, both AZO and HAZO materials exhibit mobility decrease with increasing doping levels primarily due to higher scattering probability, in particular, due to excessive ionized dopant atoms acting as scattering centers and decreasing grain sizes.^61^ For the pure ZnO sample, a 28 cm^2^/Vs value of mobility is obtained that almost linearly decreases with dopant concentration to the values of 6 and 7.5 cm^2^/Vs at 10% for AZO and HAZO, respectively. The results clearly indicate that at the same doping level, HAZO consistently achieves higher mobility than AZO, suggesting that the addition of hafnium may reduce the number of scattering centers. Besides, increasing doping leads to a significant carrier concentration rise from 2 × 19 to 3.3 × 20 cm^–3^ for AZO with 4% Al content, and 3.7 × 20 cm^–3^ for HAZO, with 6% of dopants content. It can be observed that both AZO and HAZO exhibit very alike carrier concentration increase characteristics with linear growth until a maximum at around 6% of dopant content. Increasing doping above this level results in an abrupt decrease rather than providing additional free carriers, possibly due to the generation of a significant amount of trapping states in connection with the deterioration of the crystal lattice. As it can be observed, HAZO achieves slightly higher free electron concentrations for similar doping levels, compared to AZO. Hafnium addition again has a beneficial influence on this parameter, possibly by passivating deep states responsible for trapping free electrons in the grain boundaries and thus raising the achievable carrier concentration.

**Table 1:** Electrical Parameters of the Investigated TCO Samples

material	thickness [nm]	doping [%]	*n* [cm^–3^]	μ [cm^2^/Vs]	ρ [10^–3^ Ωcm]
AZO	170	0	0.35	29.3	6.12
		1.9	1.14	21.1	2.60
		3.8	2.36	17.5	1.51
		6.1	3.25	11.1	1.72
		9.8	2.11	6.1	4.86
HAZO	170	1.9	1.11	23.7	2.37
		3.8	2.18	18.3	1.57
		6.0	3.73	13.8	1.21
		9.6	2.43	7.5	3.41
AZO	570	3.8	3.19	18.7	1.05
HAZO		6.0	4.45	14.7	0.95

The resistivity of the films exhibits a parabola-like shape, first decreasing from ca. 7 × 10^–3^ Ωcm for ZnO and reaching the minimum at 6% dopant concentration for HAZO, and at 5% dopant concentration for AZO. For higher doping, the resistivity starts to grow due to the saturation of free electron concentration and progressive decrease in mobility. The obtained resistivity minimum is at a 1.2 × 10^–3^ Ωcm level. As thin film’s resistivity tends to decrease with the thickness of the sample, two additional growths for optimized parameters for both AZO and HAZO were conducted for substantially thicker, 570 nm, samples. In this case, even lower resistivity values were obtained, equal to 1.05 × 10^–3^ and 9.5 × 10^–4^ Ωcm for AZO and HAZO samples, respectively.

### 2.4 Optical Properties

**Figure 7:**
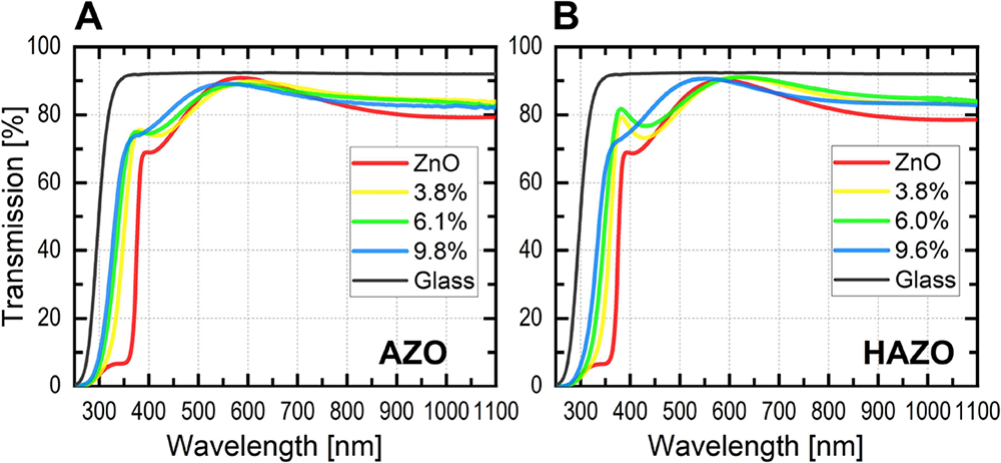
Optical transmittance spectra of (A) AZO and (B) HAZO thin films with different dopant concentrations deposited on a glass substrate (included in the graph).

**Figure 8:**
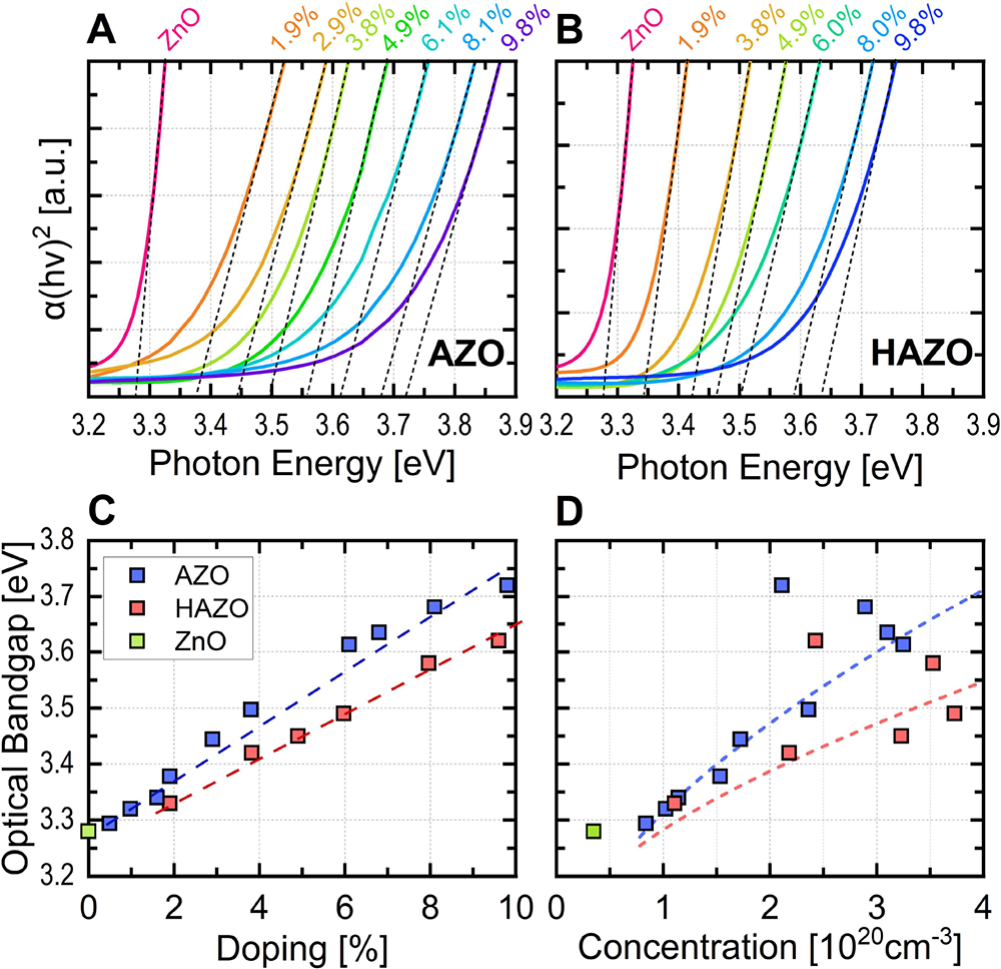
Tauc plots of (A) AZO and (B) HAZO thin films with varying dopant concentrations obtained from transmittance spectra together with linear fits pointing toward optical band gap value (dashed lines). (C) Optical band gap values determined from the Tauc plot for corresponding samples. Dashed lines are a guide to the eye. (D) Calculated band gap widening and narrowing effects. Dashed lines are calculated according to eq 2. Carrier concentrations are obtained from Hall measurements.

Transmission spectra of the selected, representative samples are shown in Figure 7 for AZO and HAZO samples with different dopant concentrations along with the glass reference spectrum. The samples regardless of the dopant type exhibit an average transmittance of 86% in the 300–1000 nm wavelength range. With increasing cation concentration, a blueshift of the absorption edge is visible due to band gap widening. This leads to the transmission increase at 400–500 nm range. To quantitatively evaluate this widening, the transmission spectra were plotted in Tauc convention, according to the following relation:
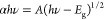
1where α is the absorption coefficient, *h* is the Planck constant, ν is the radiation frequency, and *A* is the proportionality constant. The exponent value equal to 1/2 denotes direct allowed transitions, characteristic of direct energy gap semiconductors, which is the case for ZnO. Here, the assumption that ZnO bands are approximately parabolic is implemented. This assumption is according to many reports correct for low dopant concentrations.^44,62,63^ The Tauc plots are shown in Figure 8.

The absorption edge is blue-shifted, mostly due to the Burstein–Moss effect since the free carriers of significantly increased concentration fill the lower states in the conduction band, disallowing usually available transitions. For high carrier concentrations, the band gap narrowing term should be taken into account that originates from many-body interactions, in particular, electron–electron and electron-impurity scattering effects.^64^ As many reports show,^65,66^ the deviation from the Burstein–Moss effect is increasing with dopant concentration, suggesting the amplification of those effects along with the nonparabolicity of the conduction band in ZnO. As shown in Figure 8D, the dashed lines represent the band gap shift according to the parabolic band theory expressed by the following formula:
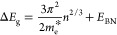
2where *m*_e_^*^ is the electron effective mass, *n* is the electron concentration, and *E*_BN_ is a band-narrowing term with nontrivial dependence on the carrier concentration and dopant ion radius. The main reasons behind the conduction band minimum lowering are both the Coulomb interaction repulsion between the electrons, which causes the effective attraction of the holes, thus shifting the energy of the band downward and electron-ion interactions. Besides, the excessive amount of electrons from donor ions in the zinc position changes the charge distribution, disrupting conduction band parabolicity. Thus, the final band gap alteration is the function of the effective mass, which itself is the function of carrier concentration.^67^ In our case, the data can be fitted using eq 2, taking into account the band-narrowing effect described above.

Monotonical widening of the band gap is exhibited by both of the materials with close to linear relation in the low doping regime. Above 8% dopant concentrations, the widening slows down. At the point for maximal free carrier concentration, the band gap widening cannot be explained solely by the carrier concentration increase, but rather by some structural changes, in particular, lattice stress from high dopant concentrations. It is widely accepted that Al (or Hf) incorporates in the lattice sites of ZnO, replacing zinc atoms with dopants that contribute 3 (or 4) additional electrons to the conduction band, at the same time suppressing oxygen vacancies formation.^68−70^ Once the lattice sites are saturated, the excessive atoms are incorporated as interstitial atoms, acting as deep defect states without providing any additional carriers.

AZO shows a slightly wider band gap opening reaching about 3.7 eV for 9% of Al doping compared to 3.62 eV for HAZO with 5% Al and 5% Hf. At their respective resistivity minima, i.e., 4 and 6% for AZO and HAZO, respectively, we observe 3.5 and 3.62 eV. As SEM and AFM studies show, the materials severely lose their crystalline quality at doping above 10%, leading to grain amorphization. The slower band gap widening observed in HAZO is intriguing, considering that higher concentration usually results in a wider band gap due to the Burstein–Moss effect. This may be explained by the presence of optically active states below the Fermi level, introduced by hafnium, which results in the lowering of the optical band gap.^42^ This effect along with many-body interactions balances the pure Burstein–Moss effect, thus causing the band gap narrowing more prominent.

### 2.5 Ellipsometry Results

**Figure 9:**
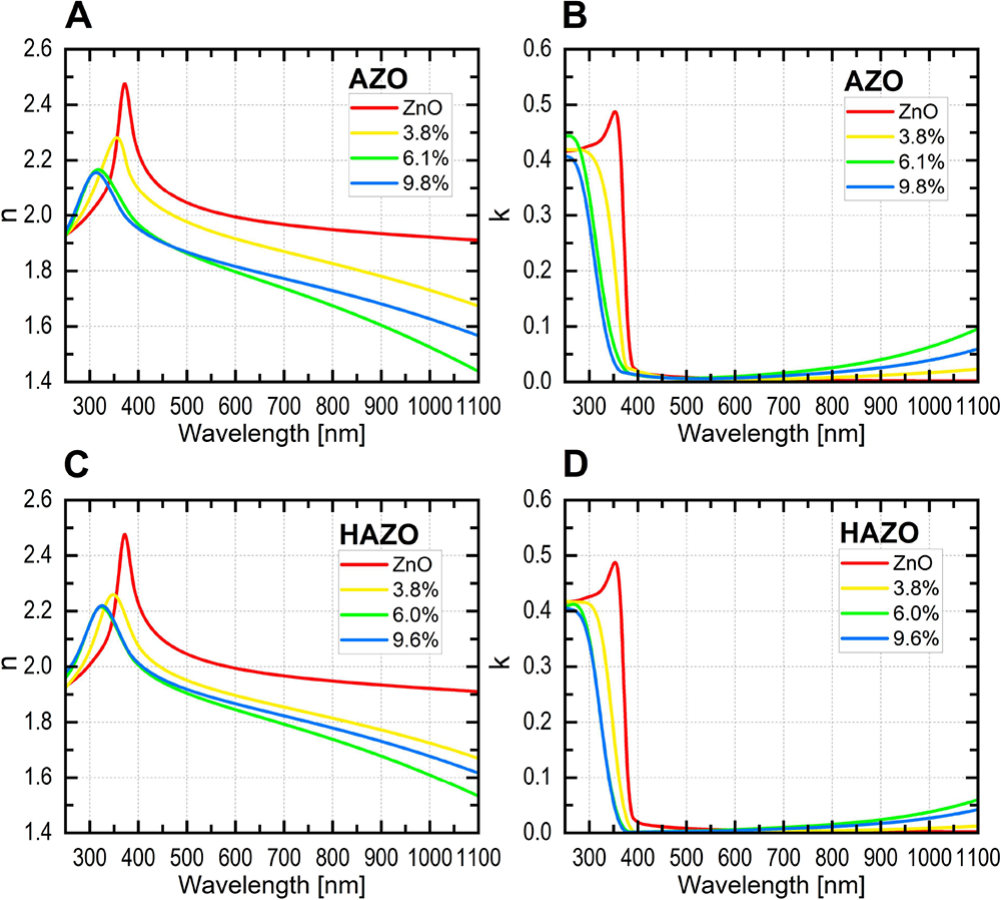
Complex dielectric functions of (A, B) AZO and (C, D) HAZO for various dopant concentrations extracted from the ellipsometric measurements. The red line refers to the optical constants of ZnO.

Figure 9 shows the real and imaginary parts of the complex refractive index *n̂* = *n* + *i* · *k* of AZO and HAZO samples depicted as a function of wavelength. In the presented spectral range, complex refractive index curves of both materials exhibit a similar behavior. Absorption occurring in the UV range due to electronic transitions results in the maximum values of the extinction coefficient k and the highest values of the real part of the refractive index n which decreases with the increase of wavelength. In the VIS–NIR range, ZnO becomes transparent (*k* ≈ 0) and the n is close to 2.

The incorporation of a dopant and increase of its concentration in AZO (HAZO) lead to the decrease of the real part of the refractive index and the shift of the UV absorption edge toward shorter wavelengths. The latter results in the blue shift of the maximum of the real part of the refractive index. Moreover, an increase of the extinction coefficient in the NIR range occurs with the increase of dopant concentration and is caused by free carrier absorption described by the Drude term as well as absorption that appears due to phonons or defect states^82^ that in the model are described by Gaussian oscillators.

Although the indicated trends are common for both AZO and HAZO materials, HAZO exhibits lower refractive index changes that are Δ*n*_HAZO_ ≈ 0.14@500 nm and Δ*n*_HAZO_ ≈ 0.31@1000 nm, while for HAZO, these changes are Δ*n*_AZO_ ≈ 0.19@500 nm and Δ*n*_AZO_ ≈ 0.41@1000 nm. The achievable refractive index range is sufficient for instance for the design of efficient Bragg gratings and other photonic components based on the optical constants modulation. Although the range of refractive index change of HAZO is smaller as compared to AZO, the extinction coefficient reaches half of the maximum value of k achievable for AZO, thus making it more transparent in the presented spectral range.

## 3 Conclusions

AZO and HAZO thin films were grown using the ALD technique under the same growth conditions with different dopant ratios using TMA and TMA with TEMAH as the cation atom precursors, respectively. These films are then thoroughly studied to gain a deeper understanding of their optoelectronic properties. The samples’ electrical and optical properties are significantly changed upon doping, in particular, they undergo conductivity enhancement. The optimal performance is achieved by codoped ZnO with approximately 3% Al and 3% Hf content. These improvements are primarily attributed to the higher free carriers concentration in the material, along with slightly larger grain lengths observed in the films with hafnium incorporated. Moreover, band gap widening due to the Burstein–Moss effect as well as band gap narrowing effects are examined. Due to additional states, the band gap widening in the case of quaternary material is slower than that for singly doped AZO samples. It appears that up to a critical doping level of approximately 8%, the dopants, regardless of their type, occupy the zinc atom sites. Once these states become saturated, the dopants begin to occupy the interstitial positions, causing expanding stress and deteriorative changes in the lattice structure.

These results provide valuable insight into the interplay between the dopants and properties of the aluminum–hafnium codoped ZnO thin films. Given the exceptional optoelectronic properties, combined with the nondemanding growth conditions and versatile tunability, the proposed material holds great promise to be a part of future electronic devices utilizing TCO films.

## 4 Methods

Thin AZO and HAZO films with various doping were deposited on glass substrates by ALD using the commercial system Beneq TFS-200. The metal precursors for Zn, Al, and Hf were diethylzinc (DEZ 99% purity), trimethylaluminum (TMA, 99% purity), and tetrakis (ethylmethylamino) hafnium (TEMAH 99% purity), respectively. Deionized water (conductivity below 1 μS) was used as the oxygen source. During the growth, H_2_O, DEZ, and TMA were kept in bottles in the liquid state at around 20 °C. TEMAH was provided via an in-built heated gas line to enhance vapor pressure, securing stable and controllable doping. The temperature of the TEMAH precursor container was set at 80 °C. The precursors were alternately supplied to the main reactor chamber using very high-purity (5 N) nitrogen carrier gas. The one cycle for ZnO was composed of 120 ms precursor pulse, followed by 8 s N_2_ purge, 120 ms H_2_O pulse, and 8 s N_2_ purge. Similarly, the doping cycle is composed of TMA or TEMAH pulse (120 ms or 500 ms, respectively) followed by 8 s N_2_ purge and 120 ms H_2_O pulse. The number of DEZ cycles between TMA and TEMAH cycles defines the final doping ratio. HAZO samples were doped symmetrically, i.e., for every one TMA cycle, there is exactly one TEMAH cycle to create equidistant doping throughout the growing sample with a 1:1 ratio of those two elements. The samples are grown using the nanolaminate approach, where the dopant precursor pulse is added periodically to achieve the desired concentration. The temperature growth was set to 200 °C and the pressure during deposition was about 4 mbar. To ensure the consistency of the reported results, we conducted transmission and electrical parameter measurements on multiple samples from each batch. Transmission spectra were collected at room temperature, with a Varian Cary 3000 photospectrometer setup in 250–1100 nm range with a 1 nm step. SEM was performed with a Carl Zeiss Sigma HV field emission microscope with the use of an In-lens detector at 15 kV accelerating voltage and around 3 mm working distance. SEM cross-section images at several representative places in a sample were used to determine the thickness of each sample. AFM was measured using Bruker DIMENSION icon with a Nanoscope 6 controller using peak force tapping. XRD measurements were performed using a Panalytical X’pert diffractometer equipped with a Cu X-ray tube. The electrical properties such as resistivity, carrier concentration, and Hall mobility were determined using Hall effect measurement utilizing the four-point van der Pauw method. The measurements were conducted at room temperature, utilizing an Ecopia HMS-3000 Hall Effect Measurement System with 0.532T neodymium built-in magnets. Uncertainties of the electrical parameters were determined based on data provided by the Ecopia HMS 3000 manufacturer, as well as the measurement setup and sample geometry. In the reported cases, the uncertainty falls below the 5% range. Ellipsometry was performed using a Woolam RC2 spectroscopic ellipsometer. The ellipsometric curves Ψ(λ) and Δ(λ), which correspond to the ratio of the reflection coefficients and the phase difference between the p- and s-polarization components of the incident beam, were recorded in the range of 193–1700 nm for the following incident angles: 45, 50, 55, 60, 65, 70, and 75°. Modeling of the optical properties was performed in CompleteEASE software. The multilayer model has taken into account all the constituents of the investigated structure, i.e., glass substrate, AZO/HAZO layer, and the roughness. Dielectric constants of glass and AZO/HAZO layers were measured directly with the ellipsometer, and the dielectric function is modeled with the use of the General Oscillator model where the real part of the permittivity is described by the ε_∞_ that is permittivity at an infinite frequency and two poles that are equivalent to a Lorentz oscillator with zero broadening, that are placed outside of the measured spectral range. The imaginary part of permittivity in the UV–VIS–IR is described by two Tauc–Lorentz oscillators in VIS and Gauss oscillators in NIR, and the metallic properties in the NIR range are parametrized by the Drude oscillator. The roughness is modeled as an effective layer assuming 50% content of voids and underlying material, the optical constants of which are calculated with the use of Bruggeman effective medium approximation.

## References

[ref1] Zhao B.; Bai S.; Kim V.; Lamboll R.; Shivanna R.; Auras F.; Richter J. M.; Yang L.; Dai L.; Alsari M.; She X. J.; Liang L.; Zhang J.; Lilliu S.; Gao P.; Snaith H. J.; Wang J.; Greenham N. C.; Friend R. H.; Di D. High-Efficiency Perovskite–Polymer Bulk Heterostructure Light-Emitting Diodes. Nat. Photonics 2018, 12, 783–789. 10.1038/s41566-018-0283-4.

[ref2] Li Y.; Meng L.; Yang Y.; Xu G.; Hong Z.; Chen Q.; You J.; Li G.; Yang Y.; Li Y. High-Efficiency Robust Perovskite Solar Cells on Ultrathin Flexible Substrates. Nat. Commun. 2016, 7, 10214 10.1038/ncomms10214. 26750664 PMC4729901

[ref3] De Wolf S.; Holovsky J.; Moon S. J.; Löper P.; Niesen B.; Ledinsky M.; Haug F. J.; Yum J. H.; Ballif C. Organometallic Halide Perovskites: Sharp Optical Absorption Edge and Its Relation to Photovoltaic Performance. J. Phys. Chem. Lett. 2014, 5, 1035–1039. 10.1021/jz500279b. 26270984

[ref4] Cho M.; Eom T.; Nundy S.; Park J. S.; Lee H. J. Conductometric Nitrogen Dioxide Gas Sensors Based on Sol-Gel-Prepared Hafnium-Added Indium Zinc Oxide (Hf-IZO). Sens. Actuators B Chem. 2021, 344, 130198 10.1016/j.snb.2021.130198.

[ref5] Kim W.; Choi M.; Yong K. Generation of Oxygen Vacancies in ZnO Nanorods/Films and Their Effects on Gas Sensing Properties. Sens. Actuators B Chem. 2015, 209, 989–996. 10.1016/j.snb.2014.12.072.

[ref6] Lewis J.; Grego S.; Chalamala B.; Vick E.; Temple D. Highly Flexible Transparent Electrodes for Organic Light-Emitting Diode-Based Displays. Appl. Phys. Lett. 2004, 85, 3450–3452. 10.1063/1.1806559.

[ref7] Levy D. H.; Freeman D.; Nelson S. F.; Cowdery-Corvan P. J.; Irving L. M. Stable ZnO Thin Film Transistors by Fast Open Air Atomic Layer Deposition. Appl. Phys. Lett. 2008, 92, 192101 10.1063/1.2924768.

[ref8] Han D. S.; Park J. H.; Kang M. S.; Choi D. K.; Park J. W. Highly Stable Hafnium-Tin-Zinc Oxide Thin Film Transistors with Stacked Bilayer Active Layers. Curr. Appl. Phys. 2015, 15, 94–97. 10.1016/j.cap.2014.11.007.

[ref9] Bohórquez C.; Bakkali H.; Delgado J. J.; Blanco E.; Herrera M.; Domínguez M. Spectroscopic Ellipsometry Study on Tuning the Electrical and Optical Properties of Zr-Doped ZnO Thin Films Grown by Atomic Layer Deposition. ACS Appl. Electron. Mater. 2022, 4, 925–935. 10.1021/acsaelm.1c01026. 35607319 PMC9121516

[ref10] Nundy S.; Ghosh A.; Tahir A.; Mallick T. K. Role of Hafnium Doping on Wetting Transition Tuning the Wettability Properties of ZnO and Doped Thin Films: Self-Cleaning Coating for Solar Application. ACS Appl. Mater. Interfaces 2021, 13, 25540 10.1021/acsami.1c04973. 34024103

[ref11] Koseoglu H.; Turkoglu F.; Kurt M.; Yaman M. D.; Akca F. G.; Aygun G.; Ozyuzer L. Improvement of Optical and Electrical Properties of ITO Thin Films by Electro-Annealing. Vacuum 2015, 120, 8–13. 10.1016/j.vacuum.2015.06.027.

[ref12] Fang M.; Zhang C.; Chen Q. Tuning the ITO Work Function by Capacitively Coupled Plasma and Its Application in Inverted Organic Solar Cells. Appl. Surf. Sci. 2016, 385, 28–33. 10.1016/j.apsusc.2016.05.077.

[ref13] Guziewicz E.; Krajewski T. A.; Przezdziecka E.; Korona K. P.; Czechowski N.; Klopotowski L.; Terziyska P. Zinc Oxide Grown by Atomic Layer Deposition: From Heavily n-Type to p-Type Material. Phys. Status Solidi B Basic Res. 2020, 257, 1900472 10.1002/pssb.201900472.

[ref14] Luka G.; Krajewski T.; Wachnicki L.; Witkowski B.; Lusakowska E.; Paszkowicz W.; Guziewicz E.; Godlewski M. Transparent and conductive undoped zinc oxide thin films grown by atomic layer deposition. Phys. Status Solidi A Appl. Mater. Sci. 2010, 207, 1568–1571. 10.1002/pssa.200983709.

[ref15] Gurylev V.; Perng T. P. Defect Engineering of ZnO: Review on Oxygen and Zinc Vacancies. J. Eur. Ceram. Soc. 2021, 41, 4977–4996. 10.1016/j.jeurceramsoc.2021.03.031.

[ref16] Gao Z.; Banerjee P. Review Article: Atomic Layer Deposition of Doped ZnO Films. J. Vac. Sci. Technol. A 2019, 37, 050802 10.1116/1.5112777.

[ref17] Lokanc M.; Eggert R.; Redlinger M. The Availability of Indium: The Present, Medium Term, and Long Term; National Renewable Energy Lab.(NREL): Golden, CO (United States), 2015. www.nrel.gov/publications.

[ref18] Zhou J.; Xu N.; Wang Z. L. Dissolving Behavior and Stability of ZnO Wires in Biofluids: A Study on Biodegradability and Biocompatibility of ZnO Nanostructures. Adv. Mater. 2006, 18, 2432–2435. 10.1002/adma.200600200.

[ref19] Özgür Ü.; Alivov Y. I.; Liu C.; Teke A.; Reshchikov M. A.; Doǧan S.; Avrutin V.; Cho S. J.; Morko H. A Comprehensive Review of ZnO Materials and Devices. J. Appl. Phys. 2005, 98, 041301 10.1063/1.1992666.

[ref20] Guziewicz E.; Godlewski M.; Wachnicki L.; Krajewski T. A.; Luka G.; Gieraltowska S.; Jakiela R.; Stonert A.; Lisowski W.; Krawczyk M.; Sobczak J. W.; Jablonski A. ALD Grown Zinc Oxide with Controllable Electrical Properties. Semicond. Sci. Technol. 2012, 27, 074011 10.1088/0268-1242/27/7/074011.

[ref21] Janotti A.; Van De Walle C. G. Fundamentals of Zinc Oxide as a Semiconductor. Rep. Prog. Phys. 2009, 72, 126501 10.1088/0034-4885/72/12/126501.

[ref22] Van De Walle C. G. Hydrogen as a Cause of Doping in Zinc Oxide. Phys. Rev. Lett. 2000, 85, 1012–1015. 10.1103/PhysRevLett.85.1012. 10991462

[ref23] Ma Y.; Du G.; Yin J.; Yang T.; Zhang Y. Structural and Optoelectrical Properties of ZnO Thin Films Deposited on GaAs Substrate by Metal-Organic Chemical Vapour Deposition (MOCVD). Semicond. Sci. Technol. 2005, 20, 1198–1202. 10.1088/0268-1242/20/12/009.

[ref24] Lany S.; Zunger A. Dopability, Intrinsic Conductivity, and Nonstoichiometry of Transparent Conducting Oxides. Phys. Rev. Lett. 2007, 98, 045501 10.1103/PhysRevLett.98.045501. 17358784

[ref25] Chen S.; Lu Z.; Zhong Z. Y.; Long H.; Gu J. H.; Long L. Microstructure and Optoelectronic Properties of Galliumtitanium-Zinc Oxide Thin Films Deposited by Magnetron Sputtering. Optoelectron. Lett. 2016, 12, 280–284. 10.1007/s11801-016-6025-2.

[ref26] Illiberi A.; Scherpenborg R.; Roozeboom F.; Poodt P. Atmospheric Spatial Atomic Layer Deposition of In-Doped ZnO. ECS J. Solid State Sci. Technol. 2014, 3, P111–P114. 10.1149/2.002405jss.

[ref27] Lee D. J.; Kwon J. Y.; Kim J.; Kim K. J.; Cho Y. H.; Cho S. Y.; Kim S. H.; Xu J.; Kim K. B. Ultrasmooth, High Electron Mobility Amorphous In-Zn-O Films Grown by Atomic Layer Deposition. J. Phys. Chem. C 2014, 118, 408–415. 10.1021/jp409738f.

[ref28] Chalker P. R.; Marshall P. A.; Romani S.; Roberts J. W.; Irvine S. J. C.; Lamb D. A.; Clayton A. J.; Williams P. A. Atomic Layer Deposition of Ga-Doped ZnO Transparent Conducting Oxide Substrates for CdTe-Based Photovoltaics. J. Vac. Sci. Technol., A 2013, 31, 01A120 10.1116/1.4765642.

[ref29] Luka G.; Wachnicki L.; Witkowski B. S.; Krajewski T. A.; Jakiela R.; Guziewicz E.; Godlewski M. The Uniformity of Al Distribution in Aluminum-Doped Zinc Oxide Films Grown by Atomic Layer Deposition. Mater. Sci. Eng. B Solid State Mater. Adv. Technol. 2011, 176, 237–241. 10.1016/j.mseb.2010.11.014.

[ref30] Elam J. W.; George S. M. Growth of Zno/Al2O3 Alloy Films Using Atomic Layer Deposition Techniques. Chem. Mater. 2003, 15, 1020–1028. 10.1021/cm020607+.

[ref31] Tao Y. M.; Ma S. Y.; Chen H. X.; Meng J. X.; Hou L. L.; Jia Y. F.; Shang X. R. Effect of the Oxygen Partial Pressure on the Microstructure and Optical Properties of ZnO:Cu Films. Vacuum 2011, 85, 744–748. 10.1016/j.vacuum.2010.11.009.

[ref32] Rekha K.; Nirmala M.; Nair M. G.; Anukaliani A. Structural, Optical, Photocatalytic and Antibacterial Activity of Zinco Xide and Manganese Doped Zinc Oxide Nanoparticles. Physica B Condens Matter 2010, 405, 3180–3185. 10.1016/j.physb.2010.04.042.

[ref33] Ahn B. D.; Kang H. S.; Kim J. H.; Kim G. H.; Chang H. W.; Lee S. Y. Synthesis and Analysis of Ag-Doped ZnO. J. Appl. Phys. 2006, 100, 093701 10.1063/1.2364041.

[ref34] Cerrato E.; Gionco C.; Berruti I.; Sordello F.; Calza P.; Paganini M. C. Rare Earth Ions Doped ZnO: Synthesis, Characterization and Preliminary Photoactivity Assessment. J. Solid State Chem. 2018, 264, 42–47. 10.1016/j.jssc.2018.05.001.

[ref35] Shukla S.; Sharma D. K. A Review on Rare Earth (Ce and Er)-Doped Zinc Oxide Nanostructures. In Materials Today: Proceedings; Elsevier Ltd, 2021; Vol. 34, pp. 793–801.

[ref36] Kim W. S.; Moon Y. K.; Kim K. T.; Shin S. Y.; Du Ahn B.; Lee J. H.; Park J. W. The Influence of Hafnium Doping on Bias Stability in Zinc Oxide Thin Film Transistors. Thin Solid Films 2011, 519, 5161–5164. 10.1016/j.tsf.2011.01.079.

[ref37] Han D. S.; Moon D. Y.; Kang Y. J.; Park J. H.; Park J. W. Improvement in the Negative Bias Stability of Zinc Oxide Thin-Film Transistors by Hafnium Doping. Curr. Appl. Phys. 2013, 13, S98–S102. 10.1016/j.cap.2013.01.004.

[ref38] Lee D. J.; Kim K. J.; Kim S. H.; Kwon J. Y.; Xu J.; Kim K. B. Atomic Layer Deposition of Ti-Doped ZnO Films with Enhanced Electron Mobility. J. Mater. Chem. C Mater. 2013, 1, 4761–4769. 10.1039/c3tc30469h.

[ref39] Bergum K.; Fjellvåg H.; Nilsen O. Thickness Dependent Structural, Optical and Electrical Properties of Ti-Doped ZnO Films Prepared by Atomic Layer Deposition. Appl. Surf. Sci. 2015, 332, 494–499. 10.1016/j.apsusc.2015.01.124.

[ref40] Qadri S. B.; Kim H.; Horwitz J. S.; Chrisey D. B. Transparent Conducting Films of ZnO-ZrO2: Structure and Properties. J. Appl. Phys. 2000, 88, 6564–6566. 10.1063/1.1320032.

[ref41] Lv M.; Xiu X.; Pang Z.; Dai Y.; Ye L.; Cheng C.; Han S. Structural, Electrical and Optical Properties of Zirconium-Doped Zinc Oxide Films Prepared by Radio Frequency Magnetron Sputtering. Thin Solid Films 2008, 516, 2017–2021. 10.1016/j.tsf.2007.06.173.

[ref42] Zhou X.; Jiang D.; Lin F.; Ma X.; Shi W. Influence of Hf Doping Concentration on Microstructure and Optical Properties of HfxZn1-XO Thin Films. Physica B Condens. Matter 2008, 403, 115–119. 10.1016/j.physb.2007.08.087.

[ref43] Kang K. M.; Wang Y.; Kim M.; Lee C.; Park H. H. Al/F Codoping Effect on the Structural, Electrical, and Optical Properties of ZnO Films Grown via Atomic Layer Deposition. Appl. Surf. Sci. 2021, 535, 147734 10.1016/j.apsusc.2020.147734.

[ref44] Zamani Meymian M. R.; Mousavi M. A.; Rabbani M.; Fallah M. Effects of Thallium–Aluminum-Codoped Zinc Oxide Thin Film as a New Transparent Conducting Oxide. Phys. Status Solidi A Appl. Mater. Sci. 2021, 218, 2000619 10.1002/pssa.202000619.

[ref45] Cao H. T.; Pei Z. L.; Gong J.; Sun C.; Huang R. F.; Wen L. S. Preparation and Characterization of Al and Mn Doped ZnO (ZnO: (Al, Mn)) Transparent Conducting Oxide Films. J. Solid State Chem. 2004, 177, 1480–1487. 10.1016/j.jssc.2003.11.030.

[ref46] Bocchese F.; Cornil D.; Haye E.; Cornil J.; Lucas S. Three-Zone Model for Ti, Al Co-Doped ZnO Films Deposited by Magnetron Sputtering. Surf. Interfaces 2022, 28, 101595 10.1016/j.surfin.2021.101595.

[ref47] Srinatha N.; Raghu P.; Mahesh H. M.; Madhu A.; Hussain S.; Dam S.; Kumar M. R.; Angadi B. Study on the Effect of Ni Co-Doping on Structural, Micro-Structural and Optical Properties of Transparent AZO Thin Films. Opt. Mater. 2021, 113, 110872 10.1016/j.optmat.2021.110872.

[ref48] Hsi C. S.; Houng B.; Hou B. Y.; Chen G. J.; Fu S. L. Effect of Ru Addition on the Properties of Al-Doped ZnO Thin Films Prepared by Radio Frequency Magnetron Sputtering on Polyethylene Terephthalate Substrate. J. Alloys Compd. 2008, 464, 89–94. 10.1016/j.jallcom.2007.10.034.

[ref49] Choi B. G.; Kim I. H.; Kim D. H.; Lee K. S.; Lee T. S.; Cheong B.; Baik Y. J.; Kim W. M. Electrical, Optical and Structural Properties of Transparent and Conducting ZnO Thin Films Doped with Al and F by Rf Magnetron Sputter. J. Eur. Ceram. Soc. 2005, 25, 2161–2165. 10.1016/j.jeurceramsoc.2005.03.023.

[ref50] El Ghoul J.; Bouguila N.; Gómez-Lopera S. A.; El Mir L. Structural and Optical Properties of Nanoparticles (V, Al) Co-Doped ZnO Synthesized by Sol-Gel Processes. Superlattices Microstruct. 2013, 64, 451–459. 10.1016/j.spmi.2013.10.018.

[ref51] Kim Y. H.; Jeong J.; Lee K. S.; Park J. K.; Baik Y. J.; Seong T. Y.; Kim W. M. Characteristics of ZnO:Al Thin Films Co-Doped with Hydrogen and Fluorine. Appl. Surf. Sci. 2010, 256, 5102–5107. 10.1016/j.apsusc.2010.03.076.

[ref52] Wang F. H.; Yang T. H. Effect of Hydrogen Doping on the Properties of Al and F Co-Doped ZnO Films for Thin Film Silicon Solar Cell Applications. Thin Solid Films 2016, 605, 64–72. 10.1016/j.tsf.2015.10.020.

[ref53] Zhu B. L.; Wang J.; Zhu S. J.; Wu J.; Wu R.; Zeng D. W.; Xie C. S. Influence of Hydrogen Introduction on Structure and Properties of ZnO Thin Films during Sputtering and Post-Annealing. Thin Solid Films 2011, 519, 3809–3815. 10.1016/j.tsf.2011.01.187.

[ref54] Bang J.; Chang K. J. Diffusion and Thermal Stability of Hydrogen in ZnO. Appl. Phys. Lett. 2008, 92, 132109 10.1063/1.2906379.

[ref55] Lee S. H.; Lee T. S.; Lee K. S.; Cheong B.; Kim Y. D.; Kim W. M. Effect of Heat Treatment of Sputter Deposited ZnO Films Co-Doped with H and Al. J. Electroceram. 2009, 23, 468–473. 10.1007/s10832-008-9497-z.

[ref56] Geng Y.; Xie Z. Y.; Yang W.; Xu S. S.; Sun Q. Q.; Ding S. J.; Lu H. L.; Zhang D. W. Structural, Optical, and Electrical Properties of Hf-Doped ZnO Films Deposited by Atomic Layer Deposition. Surf. Coat. Technol. 2013, 232, 41–45. 10.1016/j.surfcoat.2013.04.050.

[ref57] Bergum K.; Hansen P. A.; Fjellvåg H.; Nilsen O. Structural, Electrical and Optical Characterization of Ti-Doped ZnO Films Grown by Atomic Layer Deposition. J. Alloys Compd. 2014, 616, 618–624. 10.1016/j.jallcom.2014.07.177.

[ref58] Pung S. Y.; Choy K. L.; Hou X.; Shan C. Preferential Growth of ZnO Thin Films by the Atomic Layer Deposition Technique. Nanotechnology 2008, 19, 435609 10.1088/0957-4484/19/43/435609. 21832704

[ref59] Mishra S.; Przezdziecka E.; Wozniak W.; Adhikari A.; Jakiela R.; Paszkowicz W.; Sulich A.; Ozga M.; Kopalko K.; Guziewicz E. Structural Properties of Thin Zno Films Deposited by Ald under O-Rich and Zn-Rich Growth Conditions and Their Relationship with Electrical Parameters. Materials 2021, 14, 4048 10.3390/ma14144048. 34300967 PMC8307850

[ref60] Ye Z. Y.; Lu H. L.; Geng Y.; Gu Y. Z.; Xie Z. Y.; Zhang Y.; Sun Q. Q.; Ding S. J.; Zhang D. W. Structural, electrical, and optical properties of Ti-doped ZnO films fabricated by atomic layer deposition. Nanoscale Res. Lett. 2013, 8, 108 10.1186/1556-276X-8-108. 23442766 PMC3630000

[ref61] Krajewski T. A.; Dybko K.; Luka G.; Wachnicki L.; Kopalko K.; Paszkowicz W.; Godlewski M.; Guziewicz E. Analysis of Scattering Mechanisms in Zinc Oxide Films Grown by the Atomic Layer Deposition Technique. J. Appl. Phys. 2015, 118, 035706 10.1063/1.4927294.

[ref62] Sernelius B. E.; Berggren K.-F.; Jin Z.-C.; Hamberg I.; Granqvist C. G. Band-Gap Tailoring of Zno by Means of Heavy Al Doping. Phys. Rev. B 1988, 37, 10244 10.1103/PhysRevB.37.10244. 9944457

[ref63] Shokhovets S.; Gobsch G.; Ambacher O. Conduction Band Parameters of ZnO. Superlattices Microstruct. 2006, 39, 299–305. 10.1016/j.spmi.2005.08.052.

[ref64] Ye J. D.; Gu S. L.; Zhu S. M.; Liu S. M.; Zheng Y. D.; Zhang R.; Shi Y. Fermi-Level Band Filling and Band-Gap Renormalization in Ga-Doped ZnO. Appl. Phys. Lett. 2005, 86, 192111 10.1063/1.1928322.

[ref65] Banerjee P.; Lee W. J.; Bae K. R.; Lee S. B.; Rubloff G. W. Structural, Electrical, and Optical Properties of Atomic Layer Deposition Al-Doped ZnO Films. In. J. Appl. Phys. 2010, 108, 043504 10.1063/1.3466987.

[ref66] Ajimsha R. S.; Das A. K.; Singh B. N.; Misra P.; Kukreja L. M. Structural, Electrical and Optical Properties of Dy Doped ZnO Thin Films Grown by Buffer Assisted Pulsed Laser Deposition. Physica E Low Dimens. Syst. Nanostruct. 2010, 42, 1838–1843. 10.1016/j.physe.2010.02.005.

[ref67] Pisarkiewicz T.; Zakrzewska K.; Leja E. Scattering of Charge Carriers in Transparent and Conducting Thin Oxide Films with a Non-Parabolic Conduction Band. Thin Solid Films 1989, 174, 217–223. 10.1016/0040-6090(89)90892-4.

[ref68] Avadhut Y. S.; Weber J.; Hammarberg E.; Feldmann C.; Schmedtaufder Günne J. Structural Investigation of Aluminium Doped ZnO Nanoparticles by Solid-State NMR Spectroscopy. Phys. Chem. Chem. Phys. 2012, 14, 11610–11625. 10.1039/c2cp41139c. 22801707

[ref69] Nakrela A.; Benramdane N.; Bouzidi A.; Kebbab Z.; Medles M.; Mathieu C. Site Location of Al-Dopant in ZnO Lattice by Exploiting the Structural and Optical Characterisation of ZnO: Al Thin Films. Results Phys. 2016, 6, 133–138. 10.1016/j.rinp.2016.01.010.

[ref70] Ahn C. H.; Kim J. H.; Cho H. K. Tunable Electrical and Optical Properties in Composition Controlled Hf:ZnO Thin Films Grown by Atomic Layer Deposition. J. Electrochem. Soc. 2012, 159, H384–H387. 10.1149/2.026204jes.

